# Microbial Biosensors: Engineered Microorganisms as the Sensing Machinery

**DOI:** 10.3390/s130505777

**Published:** 2013-05-06

**Authors:** Miso Park, Shen-Long Tsai, Wilfred Chen

**Affiliations:** 1 Department of Chemical and Biomolecular Engineering, University of Delaware, Newark, DE 19716, USA; E-Mail: miso@udel.edu; 2 Department of Chemical Engineering, National Taiwan University of Science and Technology, Taipei 106, Taiwan; E-Mail: stsai@mail.ntust.edu.tw

**Keywords:** synthetic biology, scaffolds, genetic circuits

## Abstract

Whole-cell biosensors are a good alternative to enzyme-based biosensors since they offer the benefits of low cost and improved stability. In recent years, live cells have been employed as biosensors for a wide range of targets. In this review, we will focus on the use of microorganisms that are genetically modified with the desirable outputs in order to improve the biosensor performance. Different methodologies based on genetic/protein engineering and synthetic biology to construct microorganisms with the required signal outputs, sensitivity, and selectivity will be discussed.

## 1. Introduction

Monitoring toxins, environmental pollutants, traces of dangerous chemicals, hormones, and/or pathogens accurately and rapidly is an important task in the field of environmental stewardship, health care and homeland security, due to their non-negligible impact on the ecosystem and human society [1–6]. Compared with conventional chromatographic methodologies such as gas chromatography (GC) or high-performance liquid chromatography (HPLC), that are tedious and not portable, the use of biosensors is gaining more attention as they offer rapid and on-site/point-of-care monitoring of even trace levels of targets [2,6,7]. A biosensor is a device that combines biological molecules as the recognition element with a physical transducer and provides quantitative or semi-quantitative analytical data corresponding to the target concentration [4,7,8]. By taking advantage of the high specificity and tight interaction between biological molecules and target compounds, numerous studies have been conducted in order to develop biosensors with higher accuracy and sensitivity. These sensing devices utilize whole cells, different configurations of antibodies, DNA, RNA, enzymes or receptor proteins as the recognition elements [1–3,9]. In the presence of target analytes, biological events are converted into an electrical and/or optical signal in proportion to the target concentration. Among these biological recognition elements, enzymes are most widely used because they are highly selective. However, the need for costly and tedious purification is one limitation of constructing enzyme-based biosensors [7]. Moreover, multienzymes or cofactors are typically required to generate a measurable signal for certain targets.

Whole cells are a good alternative to enzymes since they have the benefit of low cost and improved stability comparing to enzymes or other proteins. Tedious purification could be avoided and cells can be massively produced through a simple cell culturing step and the necessary cofactors are already present inside the cells. Moreover, microbes are easy to manipulate and have better stability under harsh environments [4,7]. Live microorganisms have been employed as biosensors using different sensing techniques such as electrochemical and optical detection [10–13]. The electrochemical biosensor is a widely used technique offering high sensitivity and rapid detection. Electroactive species produced or consumed by microbes are monitored by conductimetric, amperometric, impedimetric, or potentiometric methods. Optical techniques are based on the measurement of fluorescent, luminescent, colorimetric, or other optical outputs from microorganisms [7]. Since one of the important features of these biosensors is how to link a physical signal from the recognition element to a given transducer, methods to immobilize whole cells onto the transducer represent a crucial topic in the field of biosensors. Several excellent reviews have already been published on the use of physical and chemical immobilization methods to maintain the functionality and the accessibility of whole cells towards the targets and will not be repeated here [8,9,14]. Rather, we will focus on the use of microorganisms that are genetically modified with different types of measurable signals in order to improve the biosensor performance and/or to develop biosensors for novel targets [15–17]. Different methodologies based on genetic/protein engineering and synthetic biology to construct microorganisms with the required signal outputs, sensitivity, and selectivity will be discussed.

## 2. Genetically Engineered Readouts of Microbial Sensors

In whole-cell biosensing, changes in cellular metabolism, pH, and gene expression have been quantified as a response of the sensing elements to the presence of target molecules [18–21]. Microbial auxotrophy have been used to monitor growth-limiting small molecules. For example, Pfleger *et al.* constructed an autotrophic *E. coli* strain for the detection and quantification of mevalonate, an intermediate in the biosynthesis of isoprenoids, a large class of industrially important secondary metabolites that includes flavor, fragrance, anti-oxidants, steroids, and the anti-malarial drug artemisinin [18]. Since mevalonate is a key precursor whose production must be increased in order to improve the production of isoprenoids, optimizing the level of mevalonate is important in developing recombinant strains for enhanced isoprenoid production. By deleting the native pathway for the production of isopentenyl pyrophosphate (IPP) and dimethylallyl pyrophosphate (DMAPP) that are necessary for growth, and incorporating the mevalonate-utilizing pathway (exogenous mevalonate can convert into IPP and DMAPP), the mevalonate sensor was developed to measure the concentration of mevalonate in mevalonate-producing cultures through simple growth monitoring [18]. In addition, the by-products generated from the target compounds can be used as the biosensor readout. Two early examples included the conversion of α-naphthyl acetate to α-naphthol and acetate resulting in a change in pH [19], and the hydrolysis of paraoxon, an organophosphate pesticide, to a yellow colored product, *p*-nitrophenol [22]. By expressing the enzyme γ-hexachlorocyclohexane (HCH) dehydrochlorinase from *Sphingomonas paucimobilis* that degrades lindane with the concomitant generation of hydrochloric acid in *E. coli* immobilized on a polyaniline film, a whole-cell electrochemical biosensor was developed for detection of the persistent organochlorine pesticide lindane by monitoring the change in the conductivity using pulsed amperometry [23]. This whole-cell electrochemical biosensor provides a more suitable platform for on-site monitoring of environmental samples as purified HCH dehydrochlorinase is likely to be inhibited by several physicochemical factors such as organophosphrous pesticides [23].

**Figure 1.**
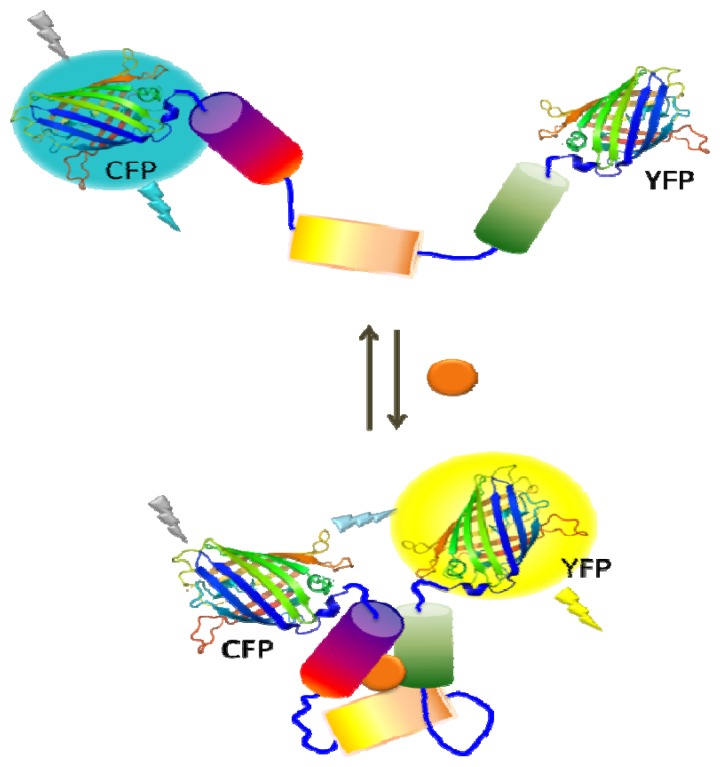
The schematic representation of the cyan fluorescent protein and yellow fluorescent protein pair and binding domains and the change of FRET upon binding of target molecules (orange).

Reporter gene expression under the control of a specific regulatory network is another powerful readout as it offers not only increased sensitivity, but also provides a simple and easy sensor platform. Commonly used reporter genes are enzymes with activities for colorimetric, fluorescent, or luminescent readouts [20,24,25]. One commonly used enzyme is β-galactosidase (β-gal) which has the advantage of detection based on either colorimetric or fluorescent methods that are simple and rapid [24]. Moreover, the availability of chemiluminescent and electrochemical substrates for β-gal offers ultrahigh sensitivity offering detection as low as 2 fg and a wide dynamic range of detection [10,26] Luciferase, which catalyzes a light-emitting reaction, is another popular enzyme for whole-cell biosensors. The bacterial luciferase catalyzes the oxidation of a reduced flavin mononucleotide (FMNH_2_) and a long-chain fatty aldehyde in the presence of molecular oxygen and results in a blue-green light emission. The primary advantages of bacterial luciferase are its sensitivity, broad dynamic range, and simplicity. Moreover, expression of whole lux operon (*luxCDABE*) has benefit of not requiring the addition of a substrate in the system [20,27]. Firefly luciferase, on the other hand, utilizes luciferin as the substrate in the presence of ATP and oxygen, resulting in the visible light emission. In addition to the high sensitivity (subattomole level) and simplicity like bacterial luciferase, firefly luciferase has the advantage of a higher quantum yield than bacterial luciferase [25,28]. Fluorescent proteins are also widely utilized in microbial sensors as there is no need to supply substrates due to their autofluorescence. In addition to its simplicity, green fluorescent protein (GFP) has been used to measure gene expression and study cell-trafficking mechanisms [29]. Fluorescent proteins of different colors such as yellow fluorescent protein (YFP), cyan fluorescent protein (CFP), and mCherry have been generated to provide the ability of simultaneous detection of multiple targets and the possibility of fluorescence resonance energy transfer (FRET) between genetically fused fluorescent protein pairs. As the efficiency of fluorescence energy transfer is dependent on the distance and orientation of two fluorescent proteins, ligand binding can be used to trigger a conformational change resulting in a change in the FRET efficiency (Figure 1). Beyond exploration of reporter enzymes for better capability, these genetically modified fluorescent proteins have been used to evaluate a wide range of target compounds [29–32].

## 3. Whole-Cell Sensor Based on Intracellular System

### 3.1. Protein: Transcriptional Regulator/Inducible Promoter Pairs

**Figure 2.**
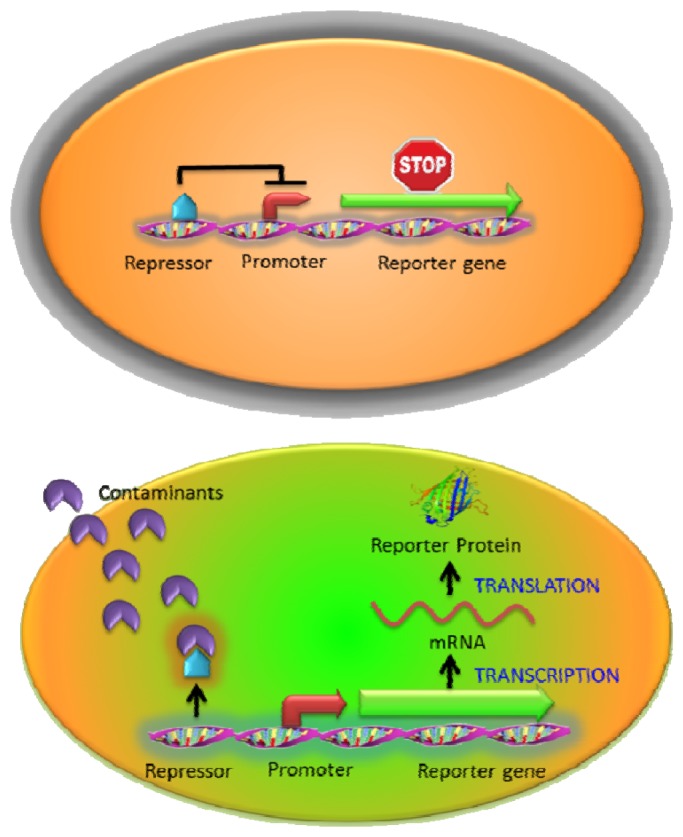
Whole cell sensing system based on the regulatory protein and inducible promoter pair. The expression of the reporter protein is repressed by binding of the regulatory protein (repressor) in the absence of analytes. In the presence of analytes, the expression of the reporter protein is induced by dissociation of repressor.

One of the most widely used intracellular sensing mechanisms is based on the coupling between a transcriptional regulator and an inducible promoter in response to different nutrient conditions, external toxicants, or communication signals [10,11]. The interaction between the target molecules and transcriptional regulators activates or represses the expression of the reporter gene resulting in a measurable signal change in a concentration-dependent manner. Most regulator protein/promoter pairs employed to detect environmental contaminants are based on the natural resistance mechanisms (such as heavy metals or antibiotics) [20,28,33] or the metabolisms of toxic compounds (such as toluene or phenol) [34,35]. In the presence of targets, native responses of the regulator and its corresponding promoter control the expression of an artificial output (reporter gene) (Figure 2). For example, a whole-cell biosensor was developed in *Bacillus subtilis* based on the regulatory protein CadC and the *cadC* promoter from *Staphylococcus sp.* for cadmium detection. The firefly luciferase genes (*lucFF*) were regulated under the *cadC* promoter and this luminescent bacterial sensor responded to cadmium (Cd), lead (Pb) and antimony (Sb) with nanomolar sensitivity [28] which was hundred to million folds more sensitive than earlier ones using bacterial luciferase [36,37]. Similarly, the ZntR regulatory protein and its corresponding *zatAp* promoter from *E. coli* were used to monitor Zn, Pb and Cd [20]. However, the whole-cell sensors stated above were not able to detect arsenic, which is far more toxic [38]. To address this issue, the arsenic detoxification *ars* operon, which is one of the most commonly exploited mechanisms for arsenic detoxification, was employed [39]. Expression from the *ars* operon is controlled by the regulatory protein ArsR through binding to the *arsR* promoter and represses the expression of reporter proteins in the absence of arsenite [40]. Upon the introduction of arsenite, the interaction between ArsR and arsenite causes the dissociation of the complex from the promoter and the reporter protein is subsequently expressed. However, earlier designs that placed the reporter genes downstream of the *arsR* gene resulted in a relatively high background due to leaky expression [39]. To reduce the background expression, Stocker *et al.* placed a transcriptional insulator, a small DNA fragment, for a second ArsR binding site downstream of the *arsR* gene and upstream of the reporter gene. Further binding of an ArsR dimer would block RNA polymerase from reading through and background expression of the reporter gene was reduced, resulting in a much lower detection limit [39].

While being a favorable feature in controlling arsenic detoxification for the cell, *ars* operon is not necessarily the most optimal for obtaining the highest sensitivity and response as a designed cellular reporter for arsenic detection. For this issue, Tani *et al.* [41] uncoupled the natural regulatory configuration by placing the expression of arsR *in trans* under control of either a *T7* or *lac* promoter while maintaining GFP expression under the control of the *arsR* promoter [41]. Recently, another group led by van der Meer systematically studied the effect of promoter strength on ArsR expression and found that a stronger constitutive ArsR production decreases arsenite-dependent EGFP output from the *ars* promoter and *vice versa* [42]. These results suggested higher expression levels and sensitivities of arsenic bioreporters using uncoupled circuits may be achieved for improving field-test assays.

For organic contaminants, the XylR and *Pu* promoter pair from the xylene degradation pathway in *Pseudomonas* have been used to detect xylene, benzene and toluene [43], and the regulatory protein TbuT and the *tbuA1p* promoter derived from the toluene degradation pathway in *Ralstonia pickettii* were used to control luciferase expression in response to these volatile compounds [41]. The regulatory protein DmpR and the *Po* promoter was used to monitor phenols in *Pseudomonas putida* with a modest detection limit of 3 μM [35], but a mutant DmpR was generated to increase the sensitivity of the biosensor by more than 4-fold than using the wild-type DmpR [44].

An *E. coli* biosensor to detect L-arabinose was developed with a detection limit of 0.5 μM and a high selectivity over D-arabinose, a stereoisomer of L-arabinose, through the AraC regulatory protein and *P_BAD_* promoter pair [45]. The AraC regulatory protein of *E. coli ara* operon represses transcription in the absence of L-arabinose but induces transcription in the presence of L-arabinose [46]. Structurally similar pentoses and hexoses including D-arabinose, cannot activate AraC and the *P_BAD_* promoter system [45]. By engineering this regulatory protein, Tang *et al.* generated a mutant AraC regulatory protein with specificity only toward D-arabinose but not L-arabinose and other sugars [47]. In addition to altering the specificity of the regulatory protein to other structurally similar compounds, efforts have been made in engineering the regulatory proteins to recognize the targets of completely different chemical structures in order to expand the flexibility of the sensing repertoire. Recently, Tang *et al.* (2011) identified a mevalonate-responsive AraC variant and employed this newly generated variant to monitor mevalonate [48]. Mevalonate is an important intracellular intermediate of the isoprenoid biosynthesis pathway and in particular, the mevalonate sensor was used as a reporter in library screening for improved mevalonate production [18]. The novel AraC variant (AraC-mev) only responded to mevalonate while there was no response observed with other chemically similar compounds L-arabinose, triacetic acid lactone, and succinate [48]. This AraC-mev variant was coupled with a *P_BAD_-LacZ* fusion to screen mutants of hydroxymethylglutaryl-CoA reductase (HMGR), one of mevalonate-dependent isoprenoid-pathway enzymes, to enhance mevalonate production as the HMGR expression level is highly sensitive to mevalonate production [48]. We think these are powerful examples demonstrating the capability to fine tune the specificity of any regulatory protein to sense a wide range of novel targets by protein engineering.

### 3.2. RNA: Riboswitch and Reporter Gene Expression

**Figure 3.**
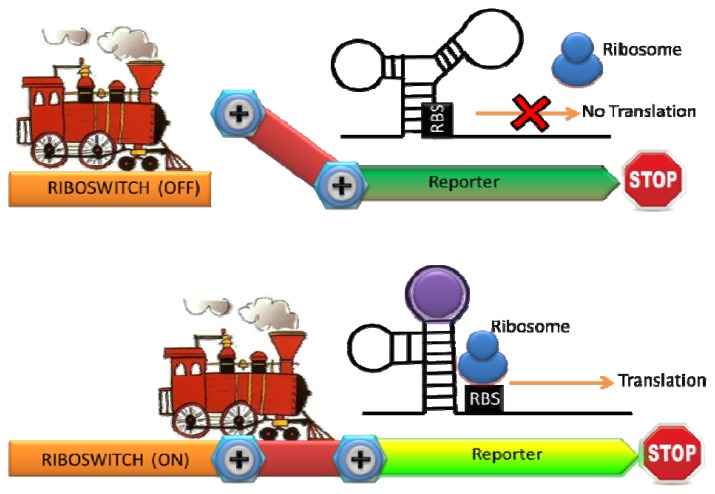
The schematic representation of the mechanism of riboswitch. The ribosome binding site (RBS) is not available for ribosome in the absence of targets. Binding of target molecules leads to the conformational shift of RNA, activating translation of the reporter protein.

Ribosomal switches are structured RNA domains which detect molecules and regulate the expression of associated genes [49]. A riboswitch is typically consisted of an aptamer domain that undergoes a conformational change upon binding to the target molecule, resulting in the expression of a reporter gene [50] (Figure 3). Since RNA is a key regulatory molecule in natural biological system and the design and engineering of RNA are relatively easy, the interest of RNA-based detection and regulation have been significantly growing last decade [51]. Riboswitches with natural and synthetic RNA aptamers have been developed to sense temperature, metal ions, nucleic acids, small molecule, and proteins [50,52–54]. A whole-cell sensor based on an engineered riboswitch was developed to detect one of the commonly used antiasthmatic drugs, theophylline [55,56]. As an overdose of theophylline leads to serious side effects, monitoring theophylline concentration in the blood serum is clinically important [57]. Thymidylate synthase, which is essential for cell growth, was linked with an anti-theophylline aptamer and the concentration of theophylline was monitored by the theophylline-dependent growth of *E. coli* [56]. To simplify the sensing format, they inserted the theophylline binding aptamer before the start codon in an engineered 5′ proximal coding region of GFP. A structural change in the 5′ proximal coding region induced by theophylline resulted in reduced ribosomal accessibility. Consequently a dose-dependent repression of GFP expression by theophylline with exquisite selectivity was observed (there was no dose-dependent repression of GFP expression by a structural analogue such as caffeine) [55].

Furthermore, an artificial riboswitch has been used for *in vivo* monitoring of intracellular metabolites and for engineering metabolic pathways [58]. This group used the natural thiamine pyrophosphate (TPP) riboswitch found in the 5′UTR of the *E. coli* thiM gene to generate a TPP-activated riboswitch. The engineered TPP-activate riboswitch was fused to β-galactosidase and GFP and the expression of these reporters was shown to induce in the presence of thiamine (which is converted to TPP intracellularly). As the interaction between the analyte and riboswitch is one of the most significant factors in determining sensitivity, a TPP riboswitch library was constructed using the TPP aptamer of the B. anthracis tenA gene with a significant higher affinity for TPP (Kd = 210 pM comparing to Kd = 600 nM of the thiM) [59]. From the library, they identified several new TPP-activated riboswitches with significantly enhanced sensitivity [59]. This result again highlights our ability to fine-tune the sensitivity by engineering the natural recognition element. Even though targets are typically limited to small molecules, the whole cell biosensors with engineered riboswitch have the potential for monitoring novel targets such as drugs and metabolites in clinical and environmental samples as riboswitch can be easily integrated into the expression platform and modulate them.

### 3.3. Quorum Sensing

Microorganisms have different cell-cell communication methods based on their lifestyles. Quorum sensing (QS), one of the most commonly used methods, is based on the use of diffusible small molecules for communication [60]. These small molecules, also known as autoinducers, are produced, secreted, and recognized via the internal biochemical pathways of the specific microorganisms. Conventionally, autoinducers have been divided into two generic groups: the acyl homoserine lactones (AHLs), used by Gram-negative bacteria and the autoinducing polypeptides (AIPs), used by Gram-positive bacteria [61]. Recently, a universal autoinducer (AI-2) was found to be used by both Gram-negative and Gram-positive bacteria [62]. When the concentration of autoinducers is beyond a defined threshold, various features such as virulence, biofilm formation, sporulation, genetic competence and bioluminescence are regulated [63]. This unique machinery has been employed in many sensing applications. Pathogens such as *Pseudomonas aeruginosa* and *Burkholderia cepacia* employ diffusible AHL signal molecules to regulate the production of a number of virulence determinants and can cause destructive lung diseases in cystic fibrosis patients [64]. Middleton and coworkers extracted sputum samples from cystic fibrosis patients colonized by either *P. aeruginosa* or *B. cepacia*, and demonstrated the clinical detection of short- and long-chain AHLs using an *E. coli* strain engineered to upregulate the expression of a reporter in response to these signal molecules [65,66]. Although this approach is capable of performing well in the laboratory, an integrated analytical device capable of on-site monitoring of pathogens would be much more practical. To adapt this sensing system into a portable format, a whole cell biosensor employing components of the AHL-mediated QS regulatory system as the recognition element and β-galactosidase as the reporter protein was liquid-dried on filter paper strips [67]. After reconstitution in LB broth for 1.5 h, the disposable biosensor was successfully applied to detect AHLs in physiological samples such as saliva at concentrations down to 1 × 10^−8^ M to correlate the presence of pathogens.

**Figure 4.**
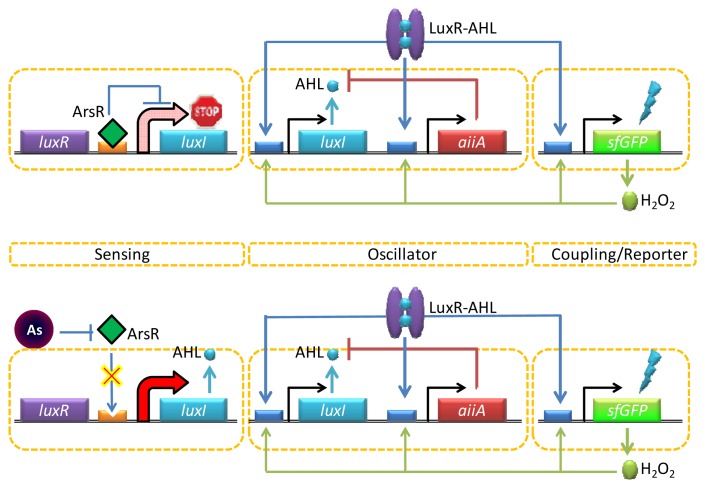
The network diagram. For the oscillator and coupling/reporter modules, the *luxI* promoter drives expression of *luxI*, *aiiA* and *sfGFP* (superfolder variant of GFP) in three identical transcription modules. The quorum-sensing genes *luxI* and *aiiA* generate synchronized oscillations within a colony while the H_2_O_2_ produced by exposing GFP to fluorescent light generate synchronized oscillations between colonies. In the sensing module, a supplementary *luxI* gene tagged for increased degradation is driven by the arsenic-responsive promoter, which affects the period of oscillation (modified from [69]).

One interesting way to enable both strong and long-range target coupling is to synchronize the response of millions of cells, and the AHL-mediated quorum sensing has recently been incorporated into this format for enhanced arsenic sensing. Quorum sensing typically involves a strong intercellular coupling over tens of micrometers [68], yet the relatively slow diffusion time of molecular communication leads to signal delays over the millimeter length scale. To overcome this challenge, Prindle *et al.* used a gene circuitry that produces an oscillating amount of GFP in an *E. coli* reporter strain maintained inside microfluidic cavities (so called ‘biopixels’) [69]. In this design, the AHL synthase LuxI is under the control of a native arsenite-responsive promoter, but the reporter gene (*gfp*) is regulated by the AHL QS system (luxR or luxI) (Figure 4). In the presence of arsenite, LuxI was expressed, and AHL was diffused across the cell membrane and mediated intercellular coupling. Interestingly, rather than taking the absolute level of GFP expression as a measure for arsenite-dependent depression of the system, this group used the duration of the oscillation period. The fast gaseous diffusion of hydrogen peroxide that is produced spontaneously as a result of oxygen radical formation at high GFP fluorescence is used to increase the production of AiiA. This in turn down-regulates the *lux* promoter controlling *gfp* expression resulting in an increase in the oscillatory amplitude and duration period depending on the arsenite concentrations. Since the oscillation period is independent of the absolute fluorescence intensity, the biopixel system output can avoid the errors coming from detector/light source fluctuations.

### 3.4. Others

Apart from the sensing mechanisms stated above, there are still several other mechanisms for the development of microbial sensors. For example, conformational changes as a result of protein-protein interactions are another way to construct live cell biosensors. Marvin *et al.* described the generation of high-signal-to-noise single wavelength biosensor for maltose [70]. By fusing the circularly permuted fluorescent proteins into a bacterial periplasmic binding protein (PBP), a maltodextrin-binding protein, the designed sensor was able to observe rapid maltose transport across the plasma membrane and the addition of maltose to extracellular media. Similarly, *E. coli* mutants with a genetically engineered glucose/galactose-binding protein (GBP) have been also used to serve as biosensors for glucose [71]. Interestingly, while the *E. coli* GBP has a high-binding affinity of 0.2 mM for glucose [72], they targeted mutants with new binding affinities within the physiological range for blood glucose monitoring [73].

While low sensitivity of live cell biosensors is always questioned by others and hinders their use, a system for developing live cell biosensors via the use of biological nanopores as sensing platforms provides the possibility toward single-molecule detection [74]. In this format, biological pores and channels that open in response to binding of individual ligands stand out as relatively simple components of single-molecule sensors [75]. In nature, single-molecule detection is routinely achieved through ligand-gated ion channel proteins [76]. A critical feature of this signaling mechanism is the strong molecular amplification that it entails. The binding of a single molecule can cause 10^4^–10^7^ ions to pass through the membrane to generate a significant change in the transmembrane potential. Using genetically engineered hemolysin pores, Bayley's group developed sensors for the detection of ionic species and organic molecules in solution [77–79].

While the systems described above are based on a known interaction between the target molecules and the bio-components, the yeast two-hybrid assay using recombinant DNA technology can be used as a biosensor to provide an important first hint for the identification of interaction partners. For the two-hybrid screening, the transcription factor is split into two parts, a binding domain and an activating domain, and the activation of downstream reporter gene(s) is achieved via the binding of a transcription factor to an upstream activating sequence by the desired interacting partners [80]. Using the two hybrid assay in yeast, interactions between the estrogen receptor and its coactivators were detected and shown to be dependent on the presence of estrogen [81]. The specificity of this assay was assessed by determining the effects of steroids, known estrogen receptor agonists, and phytoestrogens. The pattern of response to chemicals was consistent with estrogenic activity measured by other assay systems, indicating that this assay system is reliable in measuring estrogenic activity. Lee *et al.* also constructed an efficient and reliable yeast two-hybrid detection system to evaluate the estrogenic activity of potential endocrine disruptors. This system employed the interaction between the human estrogen receptor β (hERβ) ligand binding domain and the co-activator SRC1 [82].

Another elegant way for estrogen detection is via the use of chimeric proteins. These biosensors are typically constructed by fusing a target ligand-binding domain to an easily assayed reporter protein. Properly designed fusions allow ligand-induced conformational changes in the target ligand-binding domain to be transmitted to the reporter and allosterically modulate its properties. Skretas *et al.* combined the ligand-binding domains of estrogen receptors with a highly sensitive thymidylate synthase reporter for identifying diverse estrogenic compounds [83]. Expression of the reporter protein creates a hormone dependent growth phenotype in thymidylate synthase deficient *E. coli* strains. By using this sensor, they were able to rapidly screen a small chemical library and identify structurally novel estrogen receptors modulators, while predicting their agonistic/antagonistic bio-characteristics in human cell assays. This system was further employed to assay for estrogenic behavior in complex mixtures found in consumer products, including perfumes, hand and body washes, deodorants, and herbal supplements [84].

## 4. Whole-Cell Sensor Based on Extracellular System

### 4.1. Surface Display System

Surface display of peptides, proteins and epitopes on living cells has the advantage for target molecules or substrates that are not accessible to the intracellular environment. Even for targets that can be readily uptake, the overall kinetics could be still significantly improved by bypassing membrane transport. Moreover, surface display provides stabilization of enzymes and proteins through attachment to the cell wall and easy purification comparing to free proteins [19,85]. Many outer membrane proteins, lipoproteins, S-layer proteins, cell-surface appendages and autotransporters have been utilized for cell surface display. The ice nucleation protein (INP), one of outer membrane proteins of plant pathogenic bacteria, has been widely used for bacteria cell surface display [15,86–88].

Since the utilization of enzyme based biosensors is limited by the cost and the tedious work for the purification of enzymes [4,7], whole cell biosensors expressing anticipated enzymes can overcome these limitations. It has been shown that the *E. coli* cells expressing organophosphorus hydrolase (OPH) were more stable and robust than purified OPH, retaining 100% activity over a period of one month [89]. In particular, *E. coli* cells expressing OPH on the cell surface degrade parathion and paraoxon sevenfold faster than whole cells expressing OPH intracellularly [89], suggesting that reduction of the mass-transport limitation of substrates and products across the cell membrane results in consequent improvement of the overall kinetics. Our group has developed several engineered strains displaying organophosphorus hydrolase (OPH) on the cell surface for the simultaneous hydrolysis of organophosphorous pesticides into *p*-nitrophenol and its subsequent conversion into electrochemically active intermediates [15,86]. By immobilizing these engineered cells onto a carbon paste electrode, different organophosphorus pesticides can be detected by measuring the corresponding electrooxidation current generated from these intermediates [90]. Similarly, Liu *et al.* displayed xylose dehydrogenase (XDH) on the cell surface using the INP anchor for the detection of D-xylose, a compound of significant interest in genetics, human nutrition, food technology and pharmacology [87]. In that study, XDH converts D-xylose to D-xylonolactone using NAD^+^ as the cofactor and the production of NADH determined at 340 nm was used to correlate with the initial xylose concentration. The XDH-displayed bacteria showed a detection limit of 2 μM of D-xylose and a broad linear range of detection up to 900 μM. Furthermore, the XDH surface displaying system selectively detected D-xylose in real samples such as food and degraded products of lignocelluloses.

In addition to using outer membrane proteins as the anchor, proteins have been successfully presented on the cell surface through the autotransporter protein system. Autotransporter proteins are displayed on the surface of Gram-negative bacteria through secretion to the periplasm by an N-terminal signal peptide and anchored on the outer membrane by the C-terminal β-domain. Both OPH and GFP were displayed onto the surface through the *E. coli* AIDA-I (adhesion involved in diffuse adherence I) autotransporter protein and used for monitoring organophosphate compounds [91].

In addition to enzymes, human antigens were displayed on the yeast surface to detect monoclonal antibodies since the direct preparation of these mammalian proteins remains challenging and laborious [92,93]. More importantly, it is more likely to produce soluble and functional mammalian proteins in yeast with the appropriate post-translational glycosylation or proteolytic modification [94]. Tang *et al.* presented human antigens on the yeast surface via the glucoamylase secretion signal sequence and the glycosylphosphatidylinositol-anchor attachment signal sequence of the native yeast glycoprotein, α-agglutinin. The antigen-displaying yeast was used to detect monoclonal antibodies using both immunofluorescence and the enzyme-linked immunosorbent assay (ELISA). An added benefit to direct sensing is the potential application in screening positive antibody-producing hybridoma cell lines for large-scale applications [94].

### 4.2. G-Protein Coupled Receptors (GPCRs)

**Figure 5.**
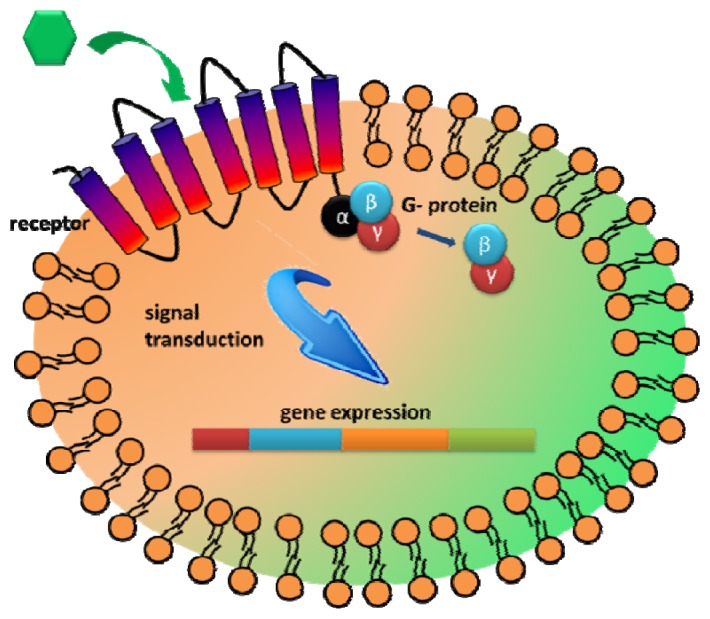
The G protein-coupled receptor located in the cell membrane binds to extracellular targets and transmits signals to an intracellular molecule, G-protein. Activation of the G protein initiates a series of intracellular reactions that ultimately result in the generation of different cellular responses.

Another interesting extracellular component that has received attention as a recognition element is the G-protein coupled receptors (GPCRs). GPCRs represent the largest family of integral membrane receptors and are involved in many intracellular communications in response to diverse external stimuli [95,96]. The primary benefit to explore GPCR is the large range of natural binding repertoire ranging from small molecules to peptides and glycoproteins [14,95,96]. As GPCRs allow transmission of a wide range of stimulants, including hormones, neurotransmitters, taste, and chemicals, they are highly modular in nature and can be customized to sense either a wide range of targets [17,96,97]. Upon ligand binding to GPCR, a signal is initiated intracellularly via interaction with the GTP-binding protein (G protein) to further transmit cellular responses [95,97] (Figure 5). Thus, the main advantage of utilizing GPCR for whole cell biosensing is that various cellular processes could be used as the sensor readout.

Although GPCR-based biosensors have been proposed for drug screening by measuring the level of secondary messengers such as GTPγS, cAMP and Ca^2+^ in mammalian cell, GPCR-based sensors have been developed by coupling with the yeast mating response pathway. Different mammalian GPCRs targeting opioids, neurotransmitters, adenosines, somatostatin or sugar nucleotides have been successfully expressed and displayed the ability of ligand binding in yeast [14,17,95,96,98]. By controlling imidazoleglycerol-phosphate dehydratase, an enzyme involved in histidine biosynthesis, under the GPCR-signaling responsive promoter, cell growth was used to correlate with the external concentration of target molecules [14,95,96,98]. Furthermore, the sensitivity and selectivity can be fine-tuned by engineering the receptors via direct evolution [17,97] and the mating responsive pathway [14,99]. For example, Broach *et al.* created a new UDP-glucose receptor by random mutagenesis and demonstrated a thirty fold lower detection limit for UDP-glucose comparing to the original receptor [17]. These sugar nucleotides are significant reagents in the biological or chemoenzymatic synthesis of carbohydrates. Since sugar nucleotides are structurally diverse and have similar physicochemical properties, they are challenging targets for inexpensive and simple high-throughput detection [17]. In addition to increasing the sensitivity by 30-fold, mutant UDP-glucose receptors with altered specificity for other sugar nucleotides have been created to extend the range of targets that can be detected [17].

## 5. Conclusions

In this review, we have highlighted recent approaches based on protein/cellular engineering and synthetic biology as an emerging tool in the field of whole-cell biosensors. In addition to engineering the sensor readouts to enhance signals, there have been continuous efforts in manipulating and generating recognition elements such as RNA, enzyme and non-enzymatic proteins with improved affinity and selectivity. Many of the approaches currently employed for improving product synthesis can easily be adapted for whole-cell biosensor design in order to optimize the sensitivity, selectivity and robustness. To use these whole-cell biosensors for rapid, point-of-care applications, developing better materials to interface with the biological elements would be of great importance.
